# Embryo movements regulate tendon mechanical property development

**DOI:** 10.1098/rstb.2017.0325

**Published:** 2018-09-24

**Authors:** Xuan Sabrina Pan, Jiewen Li, Edward B. Brown, Catherine K. Kuo

**Affiliations:** 1 Department of Biomedical Engineering, University of Rochester, Rochester, NY 14620, USA; 2 Center for Musculoskeletal Research, University of Rochester School of Medicine, Rochester, NY 14620, USA; 3 Department of Neuroscience, University of Rochester School of Medicine, Rochester, NY 14620, USA; 4 Wilmot Cancer Center, University of Rochester School of Medicine, Rochester, NY 14620, USA; 5 Department of Orthopaedics, University of Rochester School of Medicine, Rochester, NY 14620, USA

**Keywords:** embryo, tendon, mechanical properties, lysyl oxidase, cross-links, biomechanics

## Abstract

Tendons transmit forces from muscles to bones to enable skeletal motility. During development, tendons begin to bear load at the onset of embryo movements. Using the chick embryo model, this study showed that altered embryo movement frequency led to changes in elastic modulus of calcaneal tendon. In particular, paralysis led to decreased modulus, whereas hypermotility led to increased modulus. Paralysis also led to reductions in activity levels of lysyl oxidase (LOX), an enzyme that we previously showed is required for cross-linking-mediated elaboration of tendon mechanical properties. Additionally, inhibition of LOX activity abrogated hypermotility-induced increases in modulus. Taken together, our findings suggest embryo movements are critical for tendon mechanical property development and implicate LOX in this process. These exciting findings expand current knowledge of how functional tendons form during development and could guide future clinical approaches to treat tendon defects associated with abnormal mechanical loading *in utero*.

This article is part of the Theo Murphy meeting issue ‘Mechanics of development’.

## 1. Introduction

Tendons are load-bearing collagenous tissues that enable skeletal motility. Kicking depends heavily on calcaneal tendons to transmit forces from the calf muscle to the calcaneal bone. Embryo movements, such as kicking, have been implicated as critical regulators of musculoskeletal tissue development [1–8]. Deprivation of movement via treatment with decamethonium bromide (DMB) results in abnormal development of bone, meniscus and joint [1,3,4]. In contrast, 4-aminopyridine (4-AP) treatment to increase chick embryo motility increases bone growth [5,9]. Fewer studies have focused on mechanical regulation of tendon development. Specifically, paralysis of early stage (e.g. Hamburger–Hamilton stage (HH)) 24 and HH28 chick embryos with DMB leads to abnormal tendon marker expression patterns and tendon tissue morphology compared with controls [3,6]. While these important studies implicate movement as a critical regulator of tendon development, the influence of mechanical loading on the formation of a mechanically-functional tendon has not been investigated.

We previously characterized the elaboration of tendon mechanical properties from early to late chick embryo development. Using atomic force microscopy (AFM), we characterized nonlinear increases in embryonic calcaneal tendon modulus from HH28 to HH43 (approx. day 5.5–17) [10]. Interestingly, the most dramatic increases in tendon modulus occur during stages that coincide with heightened movement activity (frequency). In particular, kicking frequencies peak at HH40 and HH43 [11], the same stages when chick embryo calcaneal tendon modulus increases dramatically [10]. For reference, the chick embryo is near full term by HH45 (hatches at day 21), when the tendon is nearly ready to function as a load-bearing tissue during postnatal activities (walking, jumping, etc.). Here, we were interested in how embryo movement frequency influences mechanical property development during these critical stages of functional tendon formation [10,12].

Our earlier studies identified lysyl oxidase (LOX) to be a major regulator of tendon mechanical properties [10,12]. LOX is an enzyme that oxidatively deaminates lysine and hydroxylysine residues of collagen molecules to facilitate cross-link formation between adjacent collagen molecules [13]. Our experiments revealed that inhibition of LOX activity via β-aminopropionitrile (BAPN) reduced both LOX-mediated cross-link density and tendon modulus during tissue formation without affecting collagen content or organization. Notably, statistical analysis showed modulus correlated with cross-link density (*r*^2^ = 0.80, *p* < 0.0001) with and without perturbation of LOX activity [12]. On the basis of our previous findings, we asked in the current study whether embryo movement regulation of tendon mechanical property development may involve LOX activity.

This study aimed to understand the role of embryo movement in regulating mechanical property development of calcaneal tendons and whether LOX is involved in this process. We hypothesized that perturbation of chick embryo movement activity during later developmental stages alters LOX activity and that this leads to changes in tendon mechanical properties. Paralysis (zero movement frequency) and hypermotility (increased movement frequency) led to decreases and increases in elastic modulus, respectively. Furthermore, LOX activity was downregulated with paralysis, and inhibition of LOX during hypermotility abrogated modulus increases that occurred with hypermotility alone. Taken together, mechanical loading of tendon during development is critical for the development of mechanical properties and may require the involvement of LOX. Our findings establish new insights into the role of embryo movement in musculoskeletal tissue development and how the LOX enzyme may be involved in mechanical regulation of tendon development.

## 2. Material and methods

All materials and reagents were obtained from Thermofisher (MA, USA) unless otherwise noted.

### (a) Chick embryo culture and injections

Fertilized white leghorn eggs (University of Connecticut poultry farm) were incubated at 37°C under high humidity. For developmental characterizations, eggs were not injected. For other experiments, a single dose of sterile saline (control), 0.2% DMB (rigid paralysis), 0.2% pancuronium bromide (PB) (flaccid paralysis), 0.2% 4-AP (hypermotility) or 0.2% 4-AP and 5 mg BAPN per gram of embryo dry mass (to simultaneously induce hypermotility and inhibition of LOX activity) was injected at the air sac end of an HH43 egg and incubated for another 48 h, as we previously described [10]. DMB dosage was based on previous studies that paralysed the embryo without affecting gross development [3]. 4-AP dosage was based on previous studies that showed a single injection induced hypermotility up to 400% in HH36 to HH44 chick embryos for at least 24 h [14]. At specific timepoints, chick embryos were sacrificed by decapitation and staged on the basis of anatomical features [15]. The calcaneal tendon was homogenized in TRIzol LS reagent, snap frozen for protein and enzyme assays, or embedded for cryosectioning.

### (b) Leg explant culture

Lower limbs were isolated from anatomically staged HH43 chick embryos and cultured in growth medium (Dulbecco's Modified Eagle's Medium, 10% fetal bovine serum, 1% antibiotic/antimycotic) supplemented with saline or 0.2% DMB. Media were changed after 24 h. Calcaneal tendons were harvested for LOX activity assay after 48 h.

### (c) Total RNA isolation and reverse transcription polymerase chain reaction

Total RNA was extracted from TRIzol LS-preserved samples and reverse transcription polymerase chain reaction (RT-PCR) was performed using SuperScript III One-Step RT-PCR System with Platinum *Taq* High Fidelity DNA Polymerase (Invitrogen, CA, USA) with primer pairs for *LOX*, *LOX*-like (*LOXL*) *1* to *4*, and *18S* (table 1). Product bands in the gel were analysed using fluorescence intensity-based densitometry quantification with ImageJ (National Institutes of Health, Bethesda, MD, USA).

**Table 1.** Primer sequences.

gene	forward	reverse
*LOX*	TCGGGCGGATGTTAGAGACT	AGCTGGCGTCTAACAAGTCA
*LOXL1*	TGCTACGACACCTACAACGC	GTGGTTTTGGGCTCATGGTG
*LOXL2*	CAATTCCTTGCATCCCCAACC	TAGGGCCAGGAATGCTCAGA
*LOXL3*	AGTTGGCACACTCGTACCG	CATCTTCACACCAACGACATCCT
*LOXL4*	TGCGATGATGGCTTCGACTT	CTGGCCGTAAGTAGCACTGT
*18S*	AACGGGGCCATGATTAAGAGG	TTGCGCCGGTCCAAGAATTT

### (d) Protein and enzyme activity assays

Western blot to semi-quantitatively measure proLOX levels was performed with rabbit anti-proLOX antibody or rabbit anti-β-actin antibody (Abcam, MA, USA). ProLOX and β-actin levels were quantified by densitometry at approximately 50 kDa and approximately 40 kDa molecular bands, respectively. LOX activity of each sample was measured using a LOX activity assay kit (Abcam, MA, USA). Recombinant LOXL2 enzyme (R&D Systems, MN, USA) was used as a positive control for each assay.

### (e) Cryopreservation and cryosectioning

Calcaneal tendons of staged embryos were cryoembedded as previously described [10] and cryosectioned at 50 µm thickness.

### (f) Two-photon microscopy and image analysis

Tendon cryosections were immersed in Ca^2+^/Mg^2+^-free phosphate-buffered saline (PBS) to remove optimal cutting temperature compound (O.C.T.). A 20× water objective imaged a 220 µm × 220 µm region in the centre of tendon midsubstance in PBS. Collagen was characterized by forward second harmonic generation (F-SHG) at 800 nm excitation, and signal was collected by a photomultiplier tube using a 405DF30 filter. Background was determined with laser off. Background was subtracted from each F-SHG image using MATLAB (Mathworks, MA, USA) and then average pixel intensities of each image were calculated to assess collagen content per unit area. Our prior study showed that this measurement of collagen content correlates highly with hydroxyproline content measured with biochemical assays [12].

### (g) Atomic force microscopy

Force volume-AFM was used to characterize tendon elastic modulus using our previously established methods [10]. Briefly, tendon cryosections were immersed in PBS to remove O.C.T. A silicon nitride tip probe (approx. 20 nm radius) with a spring constant of 0.06 N m^−1^ (Bruker, CA, USA) was used on an MFP-3D AFM (Asylum Research, CA, USA) to obtain force curves in a 64 × 64 two-dimensional array over a 10 µm × 10 µm tissue region in the centre of the tendon midsubstance with a 1.0 µm indentation trigger point. The sample was indented at 6 µm s^−1^, at which tendon elastic properties were previously characterized with negligible viscous effects [10]. Elastic modulus was calculated by fitting force displacement curves at each indentation to the Hertzian model using equations adapted from our previous study [10].

### (h) Statistical analysis

For RT-PCR, tendons from both legs each from at least three embryos (*N* ≥ 3) were characterized. For western blot and LOX activity assays, tendons from both legs from at least three embryos (HH41 and earlier) or two embryos (HH42 and later) were pooled and five pools (*N* = 5) were characterized. For two-photon imaging and AFM, tendons from one leg each from at least five embryos (*N* ≥ 5) were characterized on the basis of our previous study [10]. F-SHG was used to image three non-overlapping regions (see Two-photon microscopy and image analysis section above) in the midsubstance of each tendon. AFM was used to indent one region (see AFM section above) with apparent tendon fibrillar structure in the midsubstance of each tendon. One-way ANOVA was performed for comparison of LOX family mRNA levels, proLOX levels, LOX activity levels among different developmental stages, and average stages of saline-, DMB-, 4-AP-, and 4-AP+BAPN-treated HH43 chick embryos at harvest after 48 h of treatment (*α* = 0.05). Tukey's *post hoc* test was used to perform multiple comparison analysis to evaluate the statistical differences between two specific groups at the same timepoint. Two-sample *t*-test was performed to compare elastic moduli, collagen content and LOX activity levels between treatment conditions (*α* = 0.05). All analyses were performed with Graphpad Prism v. 7.0a (La Jolla, CA, USA).

## 3. Results

### (a) Tendon elastic modulus was affected by embryo movement

**Figure 1.**
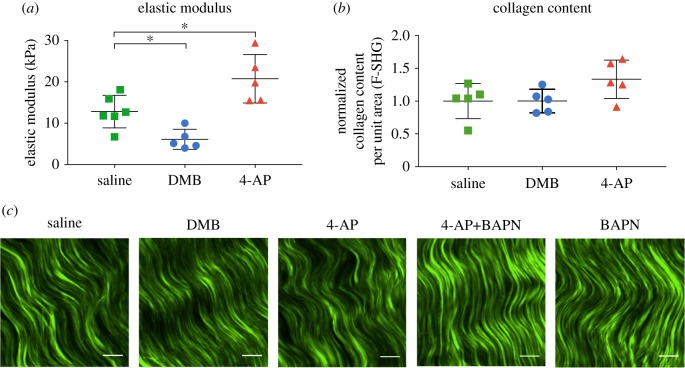
DMB and 4-AP effects on HH43 calcaneal tendons after 48 h (*N* ≥ 5). (*a*) DMB treatment led to lower elastic modulus than saline treatment. 4-AP treatment led to higher elastic modulus than saline treatment. (*b*) Normalized fibrillar collagen content (F-SHG) did not change with DMB treatment or 4-AP treatment compared with saline controls. (*c*) Representative images of fibrillar collagen detected by F-SHG for saline, DMB, 4-AP, 4-AP+BAPN and BAPN treatments. (Scale bar: 10 µm; *p < 0.05.) (Online version in colour.)

DMB treatment of HH43 chick embryos resulted in paralysis and significantly reduced calcaneal tendon modulus relative to controls (figure 1*a*). 4-AP treatment induced hypermotility and significantly upregulated tendon modulus relative to controls (figure 1*a*). Neither treatment affected collagen content or apparent organization relative to controls (figure 1*b*,*c*).

### (b) LOX levels increase during development whereas LOXL1–4 levels do not

**Figure 2.**
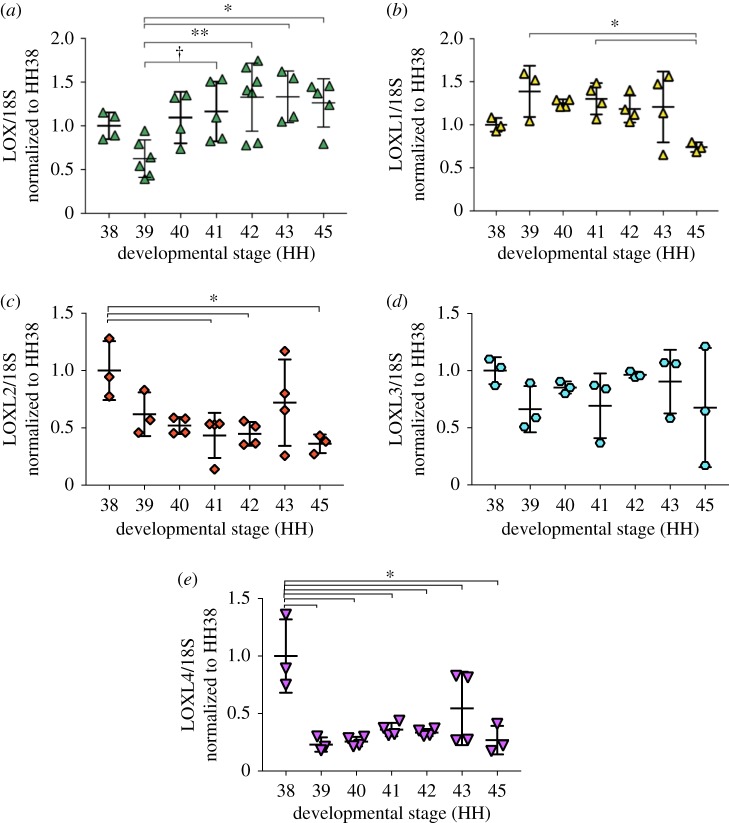
LOX and LOXL1–4 exhibited distinct gene expression profiles in developing calcaneal tendons (*N* ≥ 3). (*a*) LOX mRNA expression levels of HH41 to HH45 tendons were higher than that of HH39. (*b*) LOXL1 mRNA maintained relatively constant levels until decreasing at HH45. (*c*) LOXL2 levels decreased from HH38 to HH45. (*d*) LOXL3 levels did not change from HH38 to HH45. (*e*) LOXL4 mRNA levels decreased from HH38 to HH39 and then remained constant to HH45. (*p < 0.05; **p < 0.01; ^†^0.05 < *p* < 0.08.) (Online version in colour.)

LOX mRNA expression levels of HH41 to HH45 tendons were higher than that of HH39 (figure 2*a*). LOXL1 mRNA maintained relatively constant levels until decreasing at HH45 (figure 2*b*). LOXL2 levels decreased from HH38 to HH45 (figure 2*c*). LOXL3 mRNA levels showed no changes between stages (figure 2*d*). LOXL4 mRNA levels decreased from HH38 to HH39 and then remained constant to HH45 (figure 2*e*).

**Figure 3.**
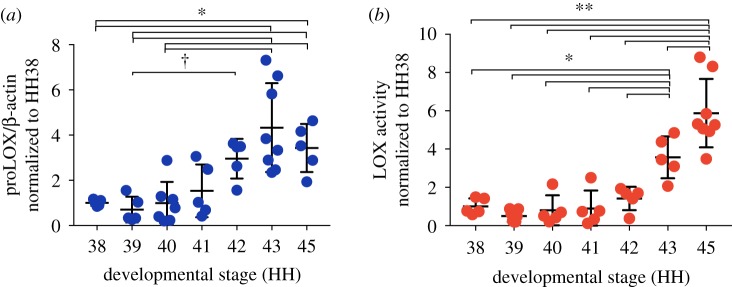
ProLOX and LOX activity levels increased in calcaneal tendons during development (*N* ≥ 5). (*a*) ProLOX levels increased from HH38 to HH42 and then plateaued. (*b*) LOX activity levels were constant from HH38 to HH42 and then increased from HH42 to HH45. (*p < 0.05; **p < 0.01; ^†^0.05 < *p* < 0.08.) (Online version in colour.)

ProLOX levels increased significantly from HH38 to HH42 and then plateaued (figure 3*a*). LOX activity levels were constant from HH38 to HH42 and then increased from HH42 to HH45 (figure 3*b*).

### (c) LOX activity levels were affected by embryo movement

**Figure 4.**
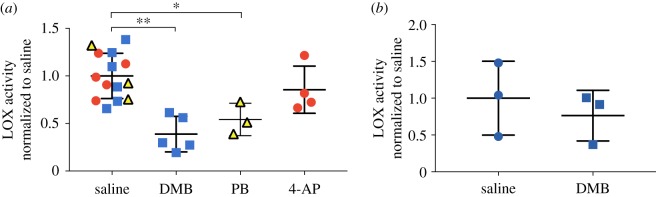
DMB and 4-AP effects on LOX activity levels of HH43 calcaneal tendons after 48 h (*N* ≥ 3). (*a*) DMB and PB treatment decreased LOX activity levels. 4-AP treatment had no effect on LOX activity levels. (*b*) DMB treatment of isolated leg explants *in vitro* had no effect on LOX activity levels. (*p < 0.05; **p < 0.01.) (Online version in colour.)

DMB treatment of HH43 embryos significantly reduced LOX activity levels in calcaneal tendons (figure 4*a*). PB treatment to induce flaccid paralysis resulted in calcaneal tendons with significantly lower LOX activity levels than controls (figure 4*a*). In contrast, 4-AP treatment had no effects on LOX activity levels compared with controls (figure 4*a*). LOX activity levels of calcaneal tendons of leg explants cultured with DMB were similar to controls (figure 4*b*).

### (d) Perturbation of LOX activity abrogated hypermotility-induced increases in modulus

**Figure 5.**
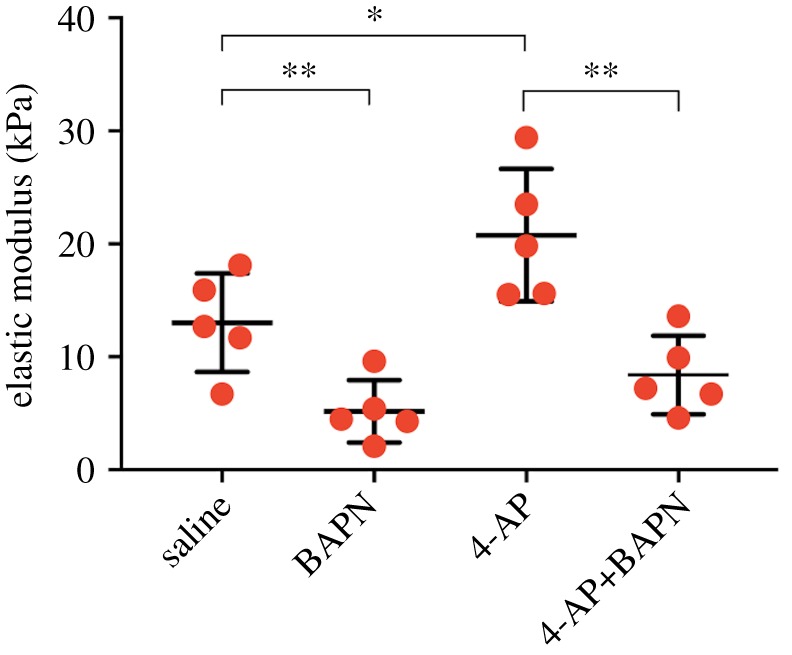
Calcaneal tendon elastic moduli of HH43 chick embryos treated for 48 h (*N* = 5). BAPN treatment decreased modulus relative to saline controls (same saline control data as in figure 1*a*); 4-AP treatment increased modulus relative to saline controls (same data as in figure 1*a*); 4-AP+BAPN treatment reduced modulus compared with 4-AP treatment alone. (*p < 0.05, **p < 0.01.) (Online version in colour.)

4-AP treatment led to higher calcaneal tendon modulus than controls (figure 5). In contrast, 4-AP+BAPN treatment resulted in significantly lower modulus compared with 4-AP treatment alone, but was similar to controls. BAPN treatment alone reduced modulus relative to controls.

### (e) Gross development was minimally affected by treatments

**Figure 6.**
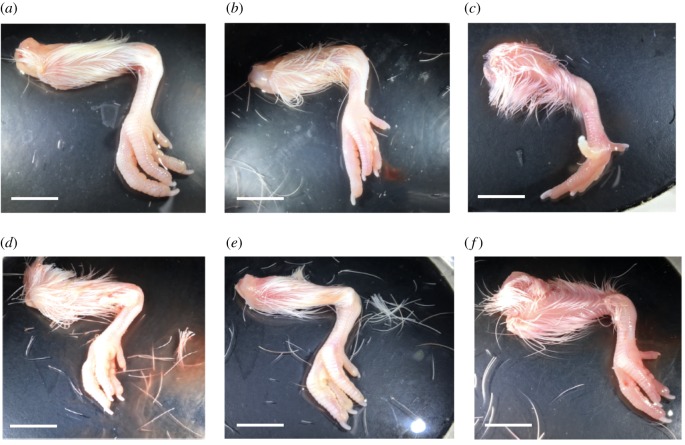
Embryos were injected at HH43, harvested after 48 h, and staged on the basis of anatomical features in comparison with non-injected HH45 embryos (*N* ≥ 6). Representative legs from (*a*) non-injected HH45 embryo; and (*b*) saline-treated; (*c*) DMB-treated, with hyperextended digits under ‘rigid paralysis’; (*d*) 4-AP-treated; (*e*) 4-AP+BAPN-treated; and (*f*) BAPN-treated HH43 embryos. (Scale bar: 10 mm; *p < 0.05.) (Online version in colour.)

HH43 chick embryos treated with saline, DMB, 4-AP and BAPN for 48 h exhibited similar anatomical features as non-injected HH45 chick embryos, reflecting normal development (table 3). In contrast, chick embryos treated with 4-AP+BAPN staged to HH44 (table 3). Grossly, legs harvested from saline-, 4-AP-, 4-AP+BAPN- and BAPN-treated embryos appeared similar to non-injected HH45 legs (figure 6*a*,*b*,*d*–*f*). DMB-treated chick embryos were still in rigid paralysis and possessed hyperextended digits at 48 h (figure 6*c*).

## 4. Discussion

Here, we tested the hypothesis that embryo movement frequency regulates tendon mechanical property development. We focused on HH43, when the embryo has formed functional muscles and tendons and is kicking at the highest frequency during development [11]. We treated HH43 chick embryos with DMB to induce paralysis (zero frequency movement), saline (normal frequency movement) and 4-AP to induce hypermotility (high frequency movement). Elastic modulus of calcaneal tendons decreased with paralysis and increased with hypermotility, relative to controls (figure 1*a*), demonstrating movement can regulate the development of tendon mechanical properties. In addition, paralysis led to reductions in LOX activity levels (figure 4*a*), implicating LOX as an important player in normal movement-regulated development of tendon mechanical properties. Changes in LOX activity were not detected after 48 h of hypermotility; however, inhibition of LOX activity during hypermotility abrogated the increases observed with hypermotility alone (figure 5), suggesting LOX may also be involved in mechanical upregulation of tendon mechanical properties. Taken together, our findings demonstrate movement critically regulates tendon mechanical property development and implicate LOX in this process.

These findings are significant because relatively little is known about the role of mechanics in tendon development. In previous studies, DMB treatment of HH24 chick embryos for 72 h diminished tendon marker expression in forelimb zeugopod and digits [6]. DMB-induced paralysis of HH35 chick embryos also led to altered tenascin-C protein distribution patterns by HH39 [3]. These studies showed that movement is important for tendon marker expression and patterning during earlier developmental stages. However, the role of mechanics in regulating tendon development at later stages had not been studied.

### (a) Tendon elastic modulus is affected by paralysis and hypermotility

DMB irreversibly binds to acetylcholine receptors in the motor end plate to trigger an immediate and permanent contraction of the muscle [7]. Consequently, DMB treatment induces a ‘rigid’ phenotype of the lower limb, effectively imposing a static (zero frequency) load on the calcaneal tendon. Here, mechanical testing of the calcaneal tendons revealed that paralysis reduced modulus by twofold relative to controls (figure 1*a*). To induce hypermotility, we treated embryos with 4-AP, a potassium channel blocker that prolongs depolarization at the neuromuscular junctions to stimulate continuous firing of muscle contraction [8]. 4-AP treatment increased embryo movement frequency by 200% within 1 min of injection, which lasted 48 h (data not shown). Mechanical testing of calcaneal tendons showed that 4-AP treatment increased modulus by twofold relative to controls (figure 1*a*). Collectively, these results suggest embryo movement is critically required for the normal development of tendon mechanical properties, and that it may be possible to enhance tendon mechanical properties by increasing movement frequency.

### (b) LOX levels increase during development but LOXL1–4 levels do not

Interestingly, collagen content and apparent collagen organization did not change with either treatment relative to controls (figure 1*b*,*c*). In earlier work, we showed inhibition of LOX activity reduces LOX-mediated collagen cross-link density and embryonic tendon modulus, without affecting collagen content or organization [10]. On the basis of these findings, we asked whether LOX is involved in the mechanically induced changes in modulus. LOX and LOXL family members have been shown to exhibit similar catalytic functions [16]. To examine which LOX family members may be involved in the development of embryonic tendon mechanical properties, we examined mRNA levels of LOX and LOXL family members (figure 2). LOXL1 to 4 levels either remained constant or decreased during development. In contrast, LOX levels increased beginning at HH39, peaked at HH42, and then plateaued to HH45. LOX was the only family member that increased during development. Because elastic modulus also increases during development, we continued to focus on LOX.

ProLOX and LOX activity levels each exhibited distinct stage-specific trends, with dramatic increases at the latest stages. Interestingly, movement frequency increases in a stage-specific manner, and increases dramatically at the latest stages [11]. Chick embryo bilateral limb movement frequency peaks at HH43 [11]. Coincidentally, LOX activity levels increased significantly from HH42 to HH43 (figure 3*b*). Notably, LOX activity levels (figure 3*b*) also correlated most highly with previously reported developing embryonic tendon moduli [10] (*r*^2^ = 0.97; *p* < 0.05) (table 2). On the basis of these data, we hypothesized that movement regulates mechanical property development of calcaneal tendon, and that these events involve LOX.

**Table 2.** Pearson's correlation between previously reported elastic moduli [10] and LOX levels (mRNA, proLOX, LOX activity) for HH38 to HH43 calcaneal tendons.

	residual value (*r*^2^)	*p*-value
LOX mRNA versus modulus	0.56	0.25
proLOX versus modulus	0.93	0.034
LOX activity versus modulus	0.97	0.016

### (c) Paralysis downregulates LOX activity

DMB treatment downregulated LOX activity levels of calcaneal tendons by twofold compared with saline controls (*p* < 0.05) (figure 4*a*), paralleling the twofold decrease in modulus (figure 1*a*). To test whether this decrease in LOX activity levels was due specifically to rigid paralysis induced by DMB, we also induced flaccid paralysis via PB treatment. PB-induced flaccid paralysis also led to twofold reductions in LOX activity levels relative to controls (*p* < 0.05) (figure 4*a*). Taken together, the two different methods to induce paralysis led to similar reductions in LOX activity levels. To confirm these results were due to paralysis and not the chemical treatment itself, we also cultured isolated legs *ex ovo* with DMB- or saline-supplemented growth medium for 48 h. No changes in tendon LOX activity levels were detected (figure 4*b*), suggesting reductions in LOX activity levels after paralysis were not due to biochemical effects of DMB. These results strongly implicate LOX as an important player in how normal embryo movement regulates the development of tendon mechanical properties.

Collagen content and organization of both DMB- and 4-AP-treated tendons appeared normal despite decreases in modulus and LOX activity levels (figure 1*b*,*c*). This was consistent with our previous study that showed inhibition of LOX activity decreases embryonic tendon moduli via reductions in LOX-mediated collagen cross-link density, and that this occurs without affecting collagen content or organization [10,12]. Perhaps DMB-induced paralysis led to decreases in LOX activity, which in turn reduced collagen cross-linking, which then led to the decrease in modulus. Future studies could use mass spectrometry to measure changes in LOX-mediated cross-link density [12].

### (d) Hypermotility-induced enhancement of tendon modulus may involve LOX

LOX activity levels did not differ between 4-AP and saline treatments (figure 4*a*). A previous study with osteoblasts detected increases in LOX activity at 18 h after treatment [17]. On the basis of this, it is possible that the 48 h timepoint we tested was too late, and we missed a window of time during which LOX activity levels were higher. To test the potential involvement of LOX with an alternative approach, we treated embryos with 4-AP+BAPN to induce hyperactivity and inhibit LOX activity simultaneously. Strikingly, 4-AP+BAPN treatment led to reductions in calcaneal tendon modulus compared with 4-AP treatment (hypermotility) alone, and was statistically similar to controls (figure 5). BAPN treatment reduced modulus, as expected [10]. Notably, despite differences in tendon elastic modulus, apparent collagen organization appeared normal after 4-AP, 4-AP+BAPN and BAPN treatments.

While these results suggest LOX could have been involved in hypermotility-induced upregulation of tendon elastic modulus, there are other possibilities. On the basis of our previous studies [10], we expected that BAPN treatment to inhibit LOX activity would reduce modulus, but this does not necessarily imply that increases in modulus with 4-AP treatment (hypermotility) also involved LOX. Instead, it is possible that 4-AP treatment increased modulus via unknown mechanisms that were unrelated to LOX, and that the BAPN effects happened to counter-balance the 4-AP effects on modulus. Alternatively, it is also possible that 4-AP-induced hypermotility led to an increase in LOX activity levels (prior to the 48 h timepoint), which led to an increase in collagen cross-linking, which led to an increase in modulus. In this case, BAPN inhibition of LOX activity may have directly abrogated hypermotility-induced increases in LOX-mediated cross-linking and modulus. In other studies, electrical stimulation of rat sciatic nerve to trigger muscle contraction increased LOX mRNA levels in Achilles tendons [18]. Treadmill running of mice abrogated BAPN-induced reductions in LOX-mediated cross-link density, modulus and yield strain of bones, relative to controls [19]. Taken together, these studies support a hypothesis that LOX played a role in the hypermotility-induced increase in tendon modulus, although future studies to test different mechanical loading modalities and perturbations will be needed.

Another observation was that 4-AP+BAPN treatment slowed the gross development of chick embryos by one stage (HH44 at harvest) compared with 4-AP treatment alone (HH45 at harvest) (table 3). However, the reductions in tendon modulus were unlikely to be due to the slight lag in development considering the elastic moduli of calcaneal tendons of 4-AP+BAPN-treated embryos were statistically similar to those of saline controls (HH45 at harvest).

**Table 3.** Average stage of chick embryos treated with saline, DMB, 4-AP, 4-AP+BAPN and BAPN at HH43 for 48 h (*N* ≥ 6). Expected stage after 48 h treatment is HH45. Statistical analysis was performed to compare average stage of each group with saline control. (*p < 0.05.)

	saline	DMB	4-AP	4-AP+BAPN	BAPN
stage (HH) of embryos at harvest (mean ± s.d.)	45 ± 0.6	45 ± 0.4	45 ± 0.4	44 ± 0.5	45 ± 0.5
*p*-value (compared with saline control)	*p* > 0.05	*p* > 0.05	*p* > 0.05	*p < 0.05	*p* > 0.05

### (e) Summary and future directions

The chick embryo is a well-established model for studying development of musculoskeletal tissues, sharing significant similarities with mammals [10,12,20–27]. Notably, the LOX amino acid sequence is highly conserved across human, mouse, rat and chicken [16]. Additionally, unlike mammals, the chick embryo can be manipulated *in ovo* and independently of maternal influences. Injecting DMB and 4-AP into the air pocket probably affected various tissues throughout the chick embryo, and it is unknown how that could have affected our results. However, the multiple perturbations in our study collectively and compellingly suggest that altered embryo movement frequency was the major contributor to changes in elastic modulus of the calcaneal tendons. Future studies could try localizing drug treatments to only the leg and test different DMB and 4-AP dosages for the effects of other kicking frequencies, although other loading parameters would not be controllable. Complementary studies using an *in vitro* bioreactor system [28–30] could impose more highly controlled strains, strain rates and frequencies on explant tendons.

Our exciting findings provide new evidence that mechanical cues are critical for embryonic tendon development. In particular, frequency of movements, such as kicking, can significantly influence tendon mechanical property elaboration and skeletal development. Additionally, our findings implicate LOX as a key player in this process. This new information could impact clinical approaches to treat musculoskeletal abnormalities that result from aberrant embryonic or fetal movements *in utero*. We also envision that greater insights into the role of mechanics in tendon development could be used to inform tendon tissue engineering and regeneration strategies.

## Data accessibility

This article has no additional data.
